# An overview of cardiovascular risk factor burden in sub-Saharan African countries: a socio-cultural perspective

**DOI:** 10.1186/1744-8603-5-10

**Published:** 2009-09-22

**Authors:** Rhonda BeLue, Titilayo A Okoror, Juliet Iwelunmor, Kelly D Taylor, Arnold N Degboe, Charles Agyemang, Gbenga Ogedegbe

**Affiliations:** 1 Department of Health Policy and Administration, 604 Ford Building, The Pennsylvania State University, University Park, PA, USA; 2 Department of Health and Kinesiology, Purdue University, Lambert Fieldhouse, West Lafeyette, Indiana, USA; 3 Department of Biobehavioral Health, The Pennsylvania State University, 315 Health and Human Development East, University Park, PA, USA; 4 Department of Medicine, Center for AIDS Prevention Studies, University of California San Francisco, 50 Beale St, San Francisco, California, USA; 5 Department of Social Medicine, Academic Medical Centre, University of Amsterdam, Amsterdam, Amsterdam, the Netherlands; 6 Department of Medicine, New York University, New York, USA

## Abstract

**Background:**

Sub-Saharan African (SSA) countries are currently experiencing one of the most rapid epidemiological transitions characterized by increasing urbanization and changing lifestyle factors. This has resulted in an increase in the incidence of non-communicable diseases, especially cardiovascular disease (CVD). This double burden of communicable and chronic non-communicable diseases has long-term public health impact as it undermines healthcare systems.

**Purpose:**

The purpose of this paper is to explore the socio-cultural context of CVD risk prevention and treatment in sub-Saharan Africa. We discuss risk factors specific to the SSA context, including poverty, urbanization, developing healthcare systems, traditional healing, lifestyle and socio-cultural factors.

**Methodology:**

We conducted a search on African Journals On-Line, Medline, PubMed, and PsycINFO databases using combinations of the key country/geographic terms, disease and risk factor specific terms such as "diabetes and Congo" and "hypertension and Nigeria". Research articles on clinical trials were excluded from this overview. Contrarily, articles that reported prevalence and incidence data on CVD risk and/or articles that report on CVD risk-related beliefs and behaviors were included. Both qualitative and quantitative articles were included.

**Results:**

The epidemic of CVD in SSA is driven by multiple factors working collectively. Lifestyle factors such as diet, exercise and smoking contribute to the increasing rates of CVD in SSA. Some lifestyle factors are considered gendered in that some are salient for women and others for men. For instance, obesity is a predominant risk factor for women compared to men, but smoking still remains mostly a risk factor for men. Additionally, structural and system level issues such as lack of infrastructure for healthcare, urbanization, poverty and lack of government programs also drive this epidemic and hampers proper prevention, surveillance and treatment efforts.

**Conclusion:**

Using an African-centered cultural framework, the PEN3 model, we explore future directions and efforts to address the epidemic of CVD risk in SSA.

## Introduction

Epidemiologic transition is associated with development and involves the process by which the pattern of mortality and disease shift. It is often characterized by a shift in communicable diseases and nutritional deficiencies to chronic diseases (non-communicable diseases (NCDs)). For example, a transformation from high infant and child mortality, episodic famine, and pre-transitional diseases related to infections to one of degenerative and chronic diseases (post-transitional diseases such as those attributed to diet, sedentary lifestyle, medical access, smoking and other behaviors i.e. cardiovascular disease (CVD), cancer, chronic lung disease and diabetes) [1-4]. According to World Health Organization (WHO) estimates, about 60% of deaths in the world are now caused by non-communicable diseases (WHO, 2002). In 2005, an estimated 17.5 million people died of CVD representing 30% of all global deaths of which 80% were from low- and middle-income countries (WHO, 2007). By 2020, studies indicate that mortality by CVD is expected to increase by 120% for women and 137% for men [5]. These findings highlight the need to explore the nature and magnitude of CVDs and other non-communicable diseases in developing countries.

Sub-Saharan Africa (SSA), consisting of those countries that are fully or partially located south of the Sahara Desert, are currently experiencing one of the most rapid epidemiological transitions characterized by increasing urbanization and changing lifestyle factors [6], which in turn have raised the incidence of NCDs, especially CVD [7]. Studies indicate that urbanization and economic development have also led to the emergence of a nutritional transition characterized by a shift to a higher caloric content diet and/or reduction of physical activity [4]. Together, these transitions create enormous public health challenges, and failure to address the problem may impose significant burden for the health sector and the economy of sub-Saharan African countries [8]

In countries such as Nigeria, Ghana and South Africa, the prevalence of chronic diseases is increasing, while the threat of communicable and poverty-related diseases (malaria, infant mortality, cholera, malnutrition) still exists [5,7,9,10]. In South Africa, CVD is the second leading cause of death after HIV accounting for up to 40% of deaths among adults [11].

This double burden of communicable and chronic NCDs has long-term public health impact as it undermines healthcare systems [5]. Sub-Saharan African countries, similar to most developing countries, often do not have the public health infrastructure and finances to address both communicable and poverty-related illness and behavior/chronic related illnesses [5]. In addition, there is reluctance on the part of health funding agencies and policy makers to divert scarce resources away from communicable diseases into other areas of disease burden, such as NCDs [9,12]. However throughout SSA, NCDs such as CVD are anticipated to soon eclipse communicable and poverty-related diseases as the leading cause of mortality and disability [13,14]. Also, evidence suggests that the increasing burden of chronic diseases has grave consequences because very few people will seek treatment, leading to high morbidity and mortality rates from potentially preventable diseases [15].

Globally, including SSA, certain risk factors have been found to account for up to 90%, of myocardial infarctions and other poor CVD outcomes such as stroke. These risk factors include smoking, alcohol consumption, obesity, diet, low physical activity, psychosocial factors, diabetes, hypertension and high lipid levels. [16].

The purpose of this paper is to explore the socio-cultural context of CVD risk prevention and treatment in SSA. We discuss risk factors specific to the sub-Saharan African context, including poverty, urbanization, developing healthcare systems, traditional healing, lifestyle and socio-cultural factors. We then present an African-centered cultural model which can be employed as an organizing framework and problem solving tool for culturally relevant interventions and programs to reduce CVD risk in SSA.

## Methods

Articles used in this overview consist of scholarly papers published between 1960 and May 2009. We conducted a search on African Journals On-Line, Medline, PubMed, and PsycINFO databases using combinations of the key country/geographic terms, disease and risk factor specific terms such as "diabetes and Congo" and "hypertension and Nigeria" (see table 1). Research articles on clinical trials were excluded from this overview. Contrarily, articles that reported prevalence and incidence data on CVD risk and/or articles that report on CVD risk-related beliefs and behaviors were included. Both qualitative and quantitative articles were included. In total, 350 articles were retrieved. However, only 126 articles met the inclusion criteria and were discussed in this overview. Also, when relevant, the definition/criteria for the CVD risk factor discussed is included in the section.

**Table 1:** Geographical and risk factor related key words

**Region/Country Specific**	Africa, sub-Saharan Africa, additionally each country in sub-Saharan Africa was also searched by name: Angola, Benin, Botswana, Burkina Faso, Burundi, Cameroon, Cape Verde, Central African Republic, Chad, Comoros, Congo, Democratic Republic of Congo, Ivory Coast, Djibouti, Equatorial Guinea, Eritrea, Ethiopia, Gabon, Gambia, Ghana, Guinea, Guinea-Bissau, Kenya, Lesotho, Liberia, Madagascar, Malawi, Mali, Mauritania, Mauritius, Mozambique, Namibia, Niger, Nigeria, Reunion, Rwanda, Sao Tome and Principe, Senegal, Seychelles, Sierra Leone Somalia, South Africa, Sudan, Swaziland, Tanzania, Togo, Uganda, Zambia, Zimbabwe
**Disease/Risk Factor Specific**	Cardiovascular disease/heart disease/heart failure, illness perceptions, stroke, hypertension/high blood pressure, salt intake, diabetes, glucose intolerance, dyslipidemia/cholesterol, smoking/tobacco and alcohol/drinking obesity/overweight/body size, physical (in)activity/exercise, diet/nutrition/food/hunger/and stress/mental health/urbanization, access to care, healthcare, culture traditional healer.

### Conditions and Risk Factors

Although the focus of this discussion is on socio-cultural aspects of CVD risk, we set the stage by providing information on the burden of common and well researched clinical risk factors in SSA, specifically hypertension, diabetes and dyslipidemia.

#### Clinical Risk Factors for CVD

According to findings from the INTERHEART study, a large global level case-control with over 29,000 cases and controls, that examined cardiovascular risk and related outcomes across continents, hypertension, diabetes and abnormal lipids are related to poor CVD outcomes; including myocardial infarction (MI) and stroke worldwide and in Africa [16].

##### Hypertension

Hypertension, once rare in West Africa, is emerging as a serious endemic threat. Hypertension has been referred to as a "silent killer" [17-19] as it often has no early detectable symptoms however it is a major cause of serious health conditions, including heart disease, stroke and renal disease [15,20]. Hypertension has been identified as a major risk factor for CVD, which has emerged as an important medical and public health issue in SSA despite the ravage being perpetuated by HIV, tuberculosis, and malaria [21-25]. Studies from various countries in SSA identify hypertension as a disease burden that requires concerted preventive and control efforts. Hypertension is defined in existing studies using either WHO criteria of blood pressure (BP) ≥ 160/95 mmHg or the JNC 7 (Joint National Committee on Prevention, Evaluation, and Treatment report) criteria of blood pressure ≥ 140/90 mmHg or self-reported antihypertensive medication use [22].

Prevalence rates for hypertension vary across and within regions in SSA. An analysis of all national data in Zimbabwe in the 1990s found that between 1990 and 1997, the national crude prevalence of hypertension increased from 1% to 4% [26]. Adedoyin and colleagues (2008) [27] found that in a semi-urban community sample of 2,097 adults, 36.6% had a BP of greater than or equal to 140/90 mmHg. A study in the Niger Delta region found the prevalence of hypertension to be 16% and 12% for males and females respectively [28]. A study in an urban area of Nigeria in the 1990s found that among more than 10,000 adults, the crude prevalence of hypertension (blood pressure > 160/95 mm Hg) was 12.4 percent with an age-adjusted rate of 7.4 percent [29]. In a prospective study conducted in rural Nigeria, the prevalence of hypertension was determined to be 7% [30].

The impact of migration from rural to urban areas was demonstrated in a longitudinal study in Kenya, in which moving from a rural to urban setting produced significant increases in BP within a short time [31]. Growing migration from rural areas to urban areas also suggest worsening prevalence of hypertension as migrants adopt lifestyle changes in physical activity, dietary habits, and stress level. Regardless of gender or type of community, advancing age is associated with an increased prevalence of hypertension [22,32], and this implies greater burden of hypertension as population aging occurs in SSA.

##### Diabetes mellitus

Diabetes was regarded as a rare disease in SSA prior to the 1990s [33]. Since the 1990s, demographic and epidemiological transitions, as well as urbanization, have rendered diabetes as one of the NCD burdens in SSA. Currently, there are 10.4 million individuals with diabetes in SSA, representing 4.2% of the global population with diabetes [34]. By 2025, it is estimated that this figure will increase by 80% to reach 18.7 million in this region, with a higher prevalence in the urban areas [14,34]. Studies indicate that an aging population, coupled with rapid urbanization, is expected to lead to the increasing prevalence of diabetes in SSA [14].

As in other parts of the world, Type 2 diabetes is more prevalent than type 1 diabetes in SSA [35]. We focus on type 2 diabetes. Studies presented define diabetes either by physician diagnosis, in-situ capillary whole blood glycemia test, or in some cases by urine or self-report. Studies listed were conducted after the WHO diabetes criteria were implemented in 1980 (modified in 1985) [36].

According to International Diabetes Federation (IDF), the current estimated prevalence rate of type 2 diabetes in Africa is about 2.8%. Countries such as Malawi and Ethiopia have rates under 2%, whereas Ghana, Sudan and South Africa have prevalence rates over 3% [37]. Regarding urban areas, the crude prevalence of type 2 diabetes ranges from 1.3% in Sudan to 6.3% in Cameroon. [38-40].

Consistent rural-urban disparities in the prevalence of type 2 diabetes have been noted in SSA with urban areas recording higher rates [33,37,41]. The crude prevalence rate of type 2 diabetes in rural communities has been found as low as <1% in rural Cameroon in 1997, 4.0% in rural Guinea in 2007 to 4.8% in rural South Africa [39,42-44]. However, in some cases such as Sudan, Elbagir (1998) [40] found no rural-urban differences.

##### Dyslipidemia

Dyslipidemia has emerged as an important CVD risk factor in SSA. For example, Norman and colleagues found that high cholesterol level (>or = 3.8 mmol/l) accounted for 59% of ischemic heart disease and 29% of ischemic stroke burden in adults age 30 and over. Studies presented in this section follow the NCEP Expert Panel on Detection Evaluation and Treatment of high blood cholesterol in Adults (ATP III) criteria. The prevalence of dyslipidemia, especially cholesterol has been shown to vary across regions in SSA.

In a study of healthy workers in Nigeria, 5% of the study population had hypercholesterolemia, 23% elevated total serum cholesterol, 51% elevated LDL-cholesterol and 60% low HDL-cholesterol, with females recording better overall lipid profiles. Population-based studies in Tanzania and Gambia also showed elevated total serum cholesterol level of >5.2 mmol/l in up to 25% of people age > 35 years [17,45]. Elevated cholesterol was more prevalent in urban than rural areas in the Gambian study. A Nigerian study among diabetics also demonstrated high prevalence of dyslipidemia among type 2 diabetics [46]. Results of a study comparing healthy people and type 2 adult diabetics showed significant association of triglycerides and HDL-cholesterol with advancing age, female gender, obesity, physical inactivity and inadequate glycemic control [47]. In a hospital study in Kenya, elevated levels of total cholesterol and triglycerides requiring therapeutic intervention were noted in type 2 diabetic patients with no obvious chronic complications [48]. While a study of more than 1,500 participants representative of rural and urban Cameroon found that hypercholesterolemia was almost non-existent where the prevalence of high cholesterol was <1% in rural areas and <3% in urban areas [49].

In a study of 248 diabetic patients attending a hospital in an urban community in Ghana, the distribution of dyslipidemia were as follows: 45% had total cholesterol above 5.2 mmol/L, 30.5% had HDL-cholesterol below 1.03 mmol/L, and 72.4% had high LDL-cholesterol. Thus, prevalence of abnormal cholesterol levels among the diabetic patients was high [50]. A community-based study of healthy adults in Port Harcourt, Nigeria, found that more than 30% of the 92 participants had elevated LDL-levels. Additionally, LDL and total cholesterol increased with increasing social-class [51]. More studies that link lipid levels to cardiovascular outcomes are needed to further establish this relationship in SSA.

### The Socio-cultural Context of CVD Risk in SSA

While understanding the burden of clinical CVD risk conditions is an important first step towards addressing the epidemic of CVD in SSA, it is also important to understand the contributing and competing socio-cultural context and related lifestyle beliefs and behaviors associated with the burden of these clinical risk factors and eventual poor cardiovascular outcomes.

#### Globalization, Socio-economic factors and CVD

For the purpose of this overview, we use the following definition of globalization developed by Chapman 2009, "a process characterized by the growing interdependence of the world's people, involves the integration of economies, culture, technologies, and governance" [52]. While globalization has resulted in many positive outcomes for SSA, such as increased access to technology, it can also have a negative effect. The blurring of geographic boundaries, urbanization, increasing gaps between rich and poor, improved transportation moving more people to urban centers and in turn decreasing physical activity, importation of other countries failures (i.e. Western/fast food), and increasing cost of health care goods such as pharmaceuticals has had a deleterious effect on the health of those in SSA [52].

Socio-economic stressors are also increasingly being recognized as major contributors to cardiovascular risk. Existing data suggests that communities in SSA currently live with a variety of psychosocial stressors including urbanization and poverty [53-56]. These stressors may significantly contribute to the rise in the burden of cardiovascular morbidity and mortality rates in SSA.

##### Poverty-related stressors

Previous studies conducted elsewhere have found that chronic poverty-related stressors, such as inadequate housing, water, sanitation, crowding, crime, air pollution, environmental conditions, low education, job insecurity, unemployment, and transportation needs, are potent predictors of poorer perceived health status [57-59]. In SSA, emerging data are beginning to show a link between some of these stressors and poor health outcomes. For example in Khayelitsha, South Africa, BeLue and colleagues (2008) [53] found that among young mothers the predictors of perceived stress include chronic poverty-related community stressors and unsupportive relations. In particular, potable water, lack of help, and unemployment of partners were found to be significant predictors of perceived stress. Another study conducted in rural Easter Cape by Mfeyana et al. (2006) [60] found that high socioeconomic deprivation, including educational level of the population, access to electricity, clean water, and refuse disposal, were consistent demographic predictors of poor health. Also in Nairobi, Kenya, Gulis et al. (2003) [61] found that environmental conditions can have major influences on health status.

##### Urbanization

One major psychosocial stressor shared by many people living in SSA is urbanization. Available literature suggests that 'the exploding growth of cities' often resulting in mega-slums in many parts of SSA may substantially lead to deterioration in the health and well-being of people due to poor quality of urban housing, sanitation issues, and limited access to efficient health care systems, as well as mobility/transportation stress [54]. Existing studies have shown that urbanization plays a significant role in increasing the burden of cardiovascular disease. For example, in a study conducted in a West African urban environment, Niakara et al. (2007) [62] found a high incidence of hypertension (40.2% in a sample of 2,087 participants) in the urban town of Ouagadougou, Burkina Faso. Sobngwi et al. (2004) [63] explored the contributions of urban-rural and socioeconomic gradients on hypertension in West Africa and found that urbanization and economic transitions were among the forces apparently driving the emergence of hypertension in West Africa. In particular, Kaufman et al. (1996) [64] found that hypertension prevalence increased across the gradient from rural farmer to urban poor to railway workers: 14, 25, and 29 percent, respectively. Several studies in South Africa found that participants who spent a longer period of their life in urban areas were more likely to be hypertensive [32] and women in particular were more likely to smoke [65].

#### Lifestyle Factors and CVD Risk

Understanding modifiable lifestyle factors such as weight status and substance use is key to making progress toward curbing the CVD epidemic in SSA.

##### Overweight/Obesity

Overweight/obesity is a major and well-known modifiable risk factor for CVD. The prevalence of overweight and obesity is growing in SSA, while the competing epidemic of malnutrition still exists [66-69]. Studies cited in this section typically use body mass index (BMI) and waist-to-hip ratios as a continuous measure or use cut-offs established by the WHO; however, proper cut-off for BMI and anthropomorphic measures may need to be established for SSA [70].

Abdominal obesity or increased waist-to-hip circumference puts one at particularly high risk for CVD. For example, in a meta-analysis of obesity among West African populations, the prevalence of obesity was 10.0% (95% CI, 6.0-15.0) [71,72]. A study in Benin [73] found that abdominal obesity was positively associated with increased probability of metabolic syndrome. Abdominal obesity also proved to be an important risk factor for heart failure among adults in Congo, where adults with increased waist-to-hip ratios had increased risk of heart failure [74].

Across many sub-Saharan African countries, obesity has been linked to both urban residence and wealth - the more wealth a person has, the more likely he or she is to be overweight or obese due to nutritional transition [73], transitions in energy expenditure due to urbanization [75] and other unknown factors [76]. Results from a study by Sobngwi et al (2004) [64], which explored the effects of lifetime exposure to an urban environment in Cameroon in relation to obesity and other cardiovascular risk factors, found that urbanization is associated with a drastic decrease in physical activity and changes in dietary habits. According to the authors, lifetime exposure to urban environment was associated with increased BMI (ρ = 0.42; P < 0.0001). Other studies have shown that obesity in rural areas is also increasing. Fezue and colleagues (2008) [77] found that over a 10-year period, there was a statistically significant increase in obesity (54% for women and 84% for men) in some rural areas in Cameroon. In urban areas, there was no significant increase in obesity rates, but there was an increase in waist circumference. Similarly, a study among rural and urban residents in Kenya found more than a 2-3-fold difference in percent overweight (approximately 40% versus 16%) obesity (approximately 16% versus 5%) among urban and rural residents respectively [78].

Throughout SSA, gender disparities exist in overweight/obesity [70,79]. Women are disproportionately affected by overweight/obesity status compared to men. The prevalence of obesity in urban West Africa more than doubled (114%) over the past decade, and this increase in prevalence was accounted for almost entirely by women [33]. In South Africa, Dugas and colleagues (2009) [80] found that among a sample of young adults in a peri-urban settlement, approximately half of the women were overweight or obese (mean BMI 31.0 kg/m); however, none of their male counter parts were overweight (mean BMI 21.6 kg/m). A study in Tanzania found that women have 4.5 the odds of being obese and are more than three times as likely to have a high waist-to-hip ratio compared to men [45].

Preferred body image may also be a factor in obesity among women in SSA. For example, a study by Holdsworth and colleagues (2004) [81] found that Senegalese women preferred overweight BMI to normal BMI. Holdsworth (2006) [82] also found that Senegalese women had adequate knowledge about obesity as a CVD risk factor yet needed additional education on the role of fruits and vegetables in reducing weight and BMI. Duda and colleagues (2007) [83] found similar results in a sample of Ghanaian women. However, another study by Duda et al in Accra, Ghana in 2006 [84] found that overweight women would be willing to reduce their body size in order to improve their health status.

Although it appears that affluence and excess consumption may cause obesity in the sub-Saharan African context, on the opposite end of the socio-economic spectrum, food insecurity may also play a role in obesity. Studies elsewhere suggest that food insecurity is positively associated with overweight in women [85]. Chaput and colleagues [86] found that food insecurity is a significant predictor (crude OR = 2.5) of over weight status (BMI > = 25) among women in Uganda but not in men. However, after accounting for socio-economic factors such as household income, food security no longer predicted overweight status, suggesting that socio-economic status may explain the relationship. In sum, women of all socio-economic strata in SSA are at risk for overweight and obesity, albeit through differing mechanisms that require further investigation.

##### Alcohol, Tobacco and CVD Risk

Substance use disorders and CVD are often comorbid. Alcohol and tobacco smoking is a risk factor for heart failure, ischemic stroke, heart disease, and acute myocardial infarction. A study by Ormel et al (2007) [87] examining the global burden of comorbid substance abuse, found that Nigerian patients with alcohol dependency were two times more likely to have comorbid heart disease compared to Nigerians who did not suffer from alcohol abuse. Similarly, in Nigerian patients being seen for heart failure treatment in a teaching hospital in Jos Nigeria, more than 24% of heart failure patients reported regular alcohol intake [88].

Alcohol consumption is also correlated with increased risk for glucose intolerance (GI) and diabetes. Puepet and colleagues (2008) [89] conducted a study to identify risk factors for type 2 diabetes in Jos, Nigeria, and found that alcohol consumption was highly prevalent in a random sample of 250 households. More than 50% of patients consumed alcohol regularly. In a study in a community dwelling of urban and rural participants in Kenya, it was found that excess alcohol consumption was related to increase likelihood of glucose intolerance by almost 4-fold (OR = 3.93, p < 0.0001) among men. This relationship did not hold for women (OR = 1.07) [90]. Gender differences in alcohol consumption have also been found in relation to heart failure. In a prospective cohort study among 320 Cameroonian adults, alcohol consumption was related to increased probability of cardiovascular death and all-cause death. Alcohol consumption was a factor for male participants (p < 0.001) but was not significant for female participants [10].

In a population sample in South Africa, cardiovascular incidents ranked second only after injuries for deaths attributable to alcohol [91]. Furthermore, Schneider and colleagues (2000) [92] showed that in South Africa, alcohol and tobacco use are related to poverty and low socio-economic position, whereas other cardiovascular risk factors such as physical inactivity are more common in wealthy populations. Overall, alcohol consumption is a risk factor for poor cardiovascular outcomes in SSA. Furthermore, it appears that gender and socio-economic position may moderate the relationship between alcohol use and CVD.

Tobacco use remains one of the most serious epidemiological risk factors in terms of prevalence of coronary artery disease [93] and smoking prevalence is increasing among men and women in SSA. A review of tobacco use and smoking research showed that males are more likely to smoke than females, and older males (age 30-49) are more likely to use tobacco products than younger males. The prevalence of smoking also increased among women with age [94]. A study by Seck et al. (2007) [95] found that among patients entering the hospital for MI treatment in Dakar, 40% of were smokers. In a hospital-based sample of 202 diabetics in Ethiopia, approximately 20% were smokers, all of whom were males [96].

In sum, men and those living in low socio-economic contexts are at increased risk for developing CVD and suffering poor CVD outcomes due to alcohol and smoking behaviors.

#### Systems Level Issues

##### Government Entities and Cardiovascular Risk Reduction

The involvement of country governments on both national levels and local jurisdictions is necessary to curb the emerging epidemic on CVD in SSA. Lack of awareness or misconceptions of cardiovascular risk factors, such as the belief that diabetes is a result of excess sugar intake, and limited knowledge of the appropriate dietary composition for a healthy diet contribute to increased CVD risk and subsequent morbidity and mortality. Lack of awareness of cardiovascular risk factors has been associated with lack of national programming for NCDs [97]. Widespread health education and awareness campaigns are needed to address these issues [98].

##### Access to Care

Despite the insurmountable cardiovascular risk burden, it is important to note that healthcare systems in many parts of Africa are designed to treat acute communicable diseases, rather than preventable NCDs [5] in part due to resources [22]. As a result, equity in terms of access to health care is constrained by the fact that patients with cardiovascular risk burden make significant demands on already scarce health resources.

The healthcare system in SSA is often challenged by lack of sufficient resources to provide adequate patient care. Both lack of institutional resources and up-to-date practical information for healthcare providers often jeopardizes patient care [99]. A review by Motala (2002) [100] noted that the increasing diabetes trends in Africa are influenced by inadequate health care infrastructure, inadequate supply of medications, and lack of available healthcare facilities and providers. Issues such as lack of protocols for diabetic complication evaluation and monitoring, little or non-existent referral systems, inadequate health facilities, and absence of multidisciplinary diabetic care teams also make diabetes care difficult [101].

Among diabetes patients in Mozambique and Zambia, patients in need of insulin were faced with the high cost of the medication when available but were also faced with lack of availability of insulin when needed [102]. Similarly, Whiting and colleagues (2003) [101] noted that the contextual, clinical, and health systems challenges to the delivery of health care for diabetes in Africa is influenced by several factors, including poor patient attendance at health clinics, short consultation time with physicians (leaving little or no time for patient education), inadequate staff, limited staff training, poor control of blood glucose and blood pressure, inadequate referral systems, and almost non-existent patient education.

Rural settings pose even a greater challenge, where there are few providers to serve the population and where distance to facilities is greater thereby increasing transportation costs [103]. Watkins et al. (2001) [104] suggest that the management of chronic disorders such as diabetes in rural African communities could be improved by decentralizing care to local village healthcare facilities to improve access to treatment and reduce mortality. This proved to be effective in improving diabetes control in a rural Ethiopian village. Watkins also suggested implementing strategies to track non-attenders in cases where healthcare is centralized to a far away location. Gil et al. (2008) [37] attributed lack of glycemic control among diabetics in rural Ethiopia to geographically scattered populations, shortage of drugs and insulin. Also, a lack of diabetes team care is a major factor behind these serious issues of diabetic control and complications.

Addo et al. (2007) [22] suggest that a significant portion of hypertension-related morbidity and mortality rates may be influenced by "low levels of detection, treatment, and control". In a hospital-based sample in Ghana, approximately 93% of hypertensive patients were non-compliant. Among those patients, 96% were non-compliant because of the cost of anti-hypertensive medication [105]. Thorogood and colleagues [106] found that in rural area of Nigeria, treatment of hypertension is often hindered by lack of medication and BP testing supplies. In many cases, traditional healers are sought due the lack of affordability and access to biomedical care and medications [107,108].

#### Traditional Healers and CVD risk

The role of traditional healing practices and practitioners in health care delivery in SSA cannot be ignored. For example, in Ghana, traditional healers have been incorporated as providers into their National Healthcare Delivery System [109,110]. Traditional and faith healers are often sought after to care for diabetes [111], hypertension [112] or adverse CVD outcomes such as stroke [113].

As stated earlier, due to cost of biomedical care and medications, traditional and faith healers often offer more accessible and affordable services. Additionally some healers offer a "cure" for diabetes or hypertension, which gives the patient the hope of eliminating any future burden related to his or her condition. For example, a study among traditional healers in the northern province of South Africa indicates that traditional and faith healers prescribe cures for diabetes patients, as opposed to treatment or management, and in fact, believe that diabetes can be reversed or cured [114,115]. It was further reported that many community health workers believe in traditional medicines and home-brewed beer as the best treatment for hypertension and that people who receive medical treatment become sicker and their health deteriorates rapidly. These healing practices are a representation of cultural beliefs, which influence health behaviors and serve as a framework for interpreting disease conditions.

#### The Intersection of Culture and CVD risks

Culture shapes health behaviours and serves as the lenses for perceiving and interpreting experiences [116-118]. Understanding the cultural framework by which disease is interpreted and managed is critical for devising lifestyle change strategies for sub-Saharan African populations. For example, interpretation of diabetes symptomology, names for diabetes and self-management of diabetes, is often interpreted through an indigenous framework. De-graft Aikins (2004) [119] found that members of the Akan ethnic group refer to diabetes as 'sugar disease' in Twi language. Similarly, Awah [120] and colleagues (2009) found that among 72 patients with diabetes, there were multiple indigenous labels for diabetes, which translate to phrases such as 'sugar, sugar sick' or illness that originates from "too much sweet things". These indigenous names also change through time. Furthermore, some participants in this sample attributed the cause of diabetes to a curse or witchcraft [120]. In a South African medical center, Kagee (2007) [121] interviewed patients with hypertension and found that patients may attribute the cause of hypertension to psychological states such as anger. Lifestyle interventions and education programs should account for local interpretations of disease origins and names in order to be effective. While some traditional practices or interpretations may seem different, examining the socio-cultural context within which such practices take place provides better insight. In addition, the role of family is essential in designing and implementing sustainable interventions in SSA.

##### The role of Family in Cardiovascular Risk Reduction

An African proverb compared the African family to a forest: "If you are outside, it is dense, if you are inside you see that each tree has its own position" (Akan, Ghana). This description is accurate in reflecting the role of the family in health decisions and behaviors. Airhihenbuwa [122] wrote that a person's identity is affirmed by his or her family, and one acting as an individual within the African context is a *no person*.

Research has shown that individual-based approaches to behaviour change are inadequate in non-Western contexts, such as Africa [123,124]. Therefore behavior changes interventions aimed at reducing cardiovascular risk factors should consider the role of culture and family in behavior change support for at-risk family members. As discussed below, one area in which the role of the family is essential to intervention efforts is effective dietary management (food intake).

##### Food and Culture in Cardiovascular Risk Reduction

Nutrition is essential in effective management of CVDs. Unfortunately, much of the research on food intake within the African context has focused extensively on nutritional components [125], dietary intakes, [126] poverty, and urbanization [62,63], without any attention to the socio-cultural context of food intake. Similar to other cultural activities in the African context, food intake is a cultural activity that goes beyond the physical consumption to defining relationships and cultural identity. Fajans (1988) [127], described food as having "transformative value" because it serves as an agent in generating, enacting, and perpetuating social and cultural processes. For example, among the aLunda in Zaire [128] kinship relations are often expressed through metaphors of eating. Food (either cooking or sharing, [129]) becomes an important way to contextualize relations and connectedness in a culture in ways that could inform sustainable intervention beyond the physical ingestion.

In line with this, intervention efforts should be sensitive to the socio-cultural contexts of communities, as "'seen" through the lenses of the community members and anchored in the realities of the communities. Cultural models, such as the PEN-3 model, allow researchers to assess the various factors that impact cardiovascular health, thereby "seeing" the health issue through the community lenses, and intervene, if necessary at multiple entry points. The PEN-3 model was developed as a thinking tool-kit in addressing health behaviors of people of the African descent and has been used in various prevention and intervention efforts [123]. By outlining an approach that examines health beliefs, decisions and behaviors within the context of culture, the model seeks to empower communities through their intrinsic positive and unique qualities so that culturally appropriate interventions can be planned, implemented and evaluated [122,123,130]. The model stresses the importance of involving the community, culture and people of interest in the dialogue, otherwise change will not be sustainable.

**Figure 1:**
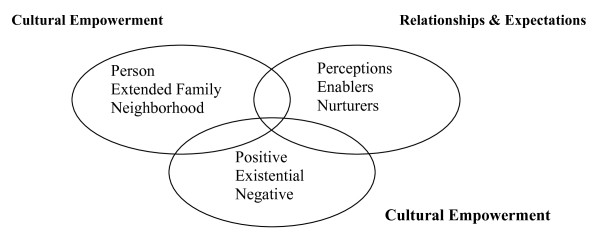
**The PEN-3 Model**.

The PEN-3 model consists of three interrelated domains; relationships and expectations, cultural empowerment, and cultural identity. Each of the domains consists of three components (see Figure 1). The first two domains, relationships and expectations, and cultural empowerment serve as the assessment tool-kit to inform the intervention, while the last domain, cultural identity, determines the point of entry or entries for intervention. In assessing the nature of the health issue and the socio-cultural context of the community, the first two domains are cross-tabulated in a 3 × 3 table (see Table 2 for an example of food intake/choices assessment). This is to ensure that the intervention is in harmony with the practices of the people, thereby increasing its effectiveness. Then, using the last domain, cultural identity, the decision is made regarding the intervention point of entry or entries: person, extended family and/or neighborhood (i.e. community including entities such as the health care system). The idea is that the intervention should not focus on just the individual but rather on the context and community within the person functions.

**Table 2:** Cross-tabulation of Relationships/Expectations and Cultural Empowerment domains exploring factors that influence dietary intake/choices.

	**Positive**	**Existential**	**Negative**
Perceptions	Food brings families together, & community building	Sharing/Eating together	Salty food tastes better; preparation techniques that remove nutrients or add excess fat

Enablers	Money to buy food Availability of healthy foods	Cooking together	Lack of money for or access healthy foods. Westernized diet in urban settings.

Nurturers	Family members support eating of food needed to manage CVD risks		Family members' refusal to eat food that supports CVD health.

The PEN-3 model provides a framework for researchers to understand the dynamics of CVD within African context. Sub-Saharan Africa is enormously diverse in people (ethnic and racial groups), culture, and socio-economic conditions. Since the PEN-3 model serves as a thinking tool to address issues among diverse contexts and cultural settings, the model can be used as a way to inform interventions and policies for CVD risk treatment and prevention in a variety of local SSA contexts.

## Concluding Comments

This paper discusses the relation between socio-cultural factors and CVD risk. The epidemic of CVD in SSA is driven by multiple factors working collectively. Lifestyle factors such as diet and smoking contribute to the increasing rates of CVD in SSA. Some lifestyle factors are considered gendered in that some are salient for women and others for men. For instance, obesity is a predominant risk factor for women compared to men, but smoking still remains mostly a risk factor for men. Additionally, structural and system level issues such as lack of infrastructure for healthcare, urbanization, poverty and lack of government programs also drive this epidemic and hampers proper prevention, surveillance and treatment efforts. Furthermore, cultural interpretations of illness may affect care seeking and management.

This paper also has several limitations. Again given the diversity of SSA, we cannot generalize our comments to all of SSA. Although we provide a general overview of socio-cultural issues and CVD risk, the relation between the two may differ among the varying cultures and contexts in SSA. Also, many SSA countries are not represented in the current literature on CVD risk. Where data exists, there is limited information, or research studies on cardiovascular disease and related risk factors.

Increased surveillance efforts and research to further illuminate the etiology, sociology and epidemiology of cardiovascular risk and disease in SSA is needed. While we recognize that ongoing surveillance and data collection is necessary to monitor the epidemic, research alone will not suffice. The development of strategies, programs and policies for reducing cardiovascular risk in order to prevent new cases of CVD and worsening of current cases is urgent. Policy, public health and health care efforts to curb this epidemic may be enhanced by incorporating a socio-cultural approach.

## Competing interests

The authors declare that they have no competing interests.

## Authors' contributions

RB conducted/conceptualized the approach and literature search, devised the search strategy, drafted the manuscript, and supervised manuscript preparation. TAO conducted the literature search and drafted the manuscript. JI conducted the literature search and drafted the manuscript. KDT conducted the literature search and contributed to drafting and editing the manuscript. AD conducted the literature search and drafted parts of the manuscript. CA and GO contributed to the editing of the manuscript. All authors read and approved the final manuscript.

## References

[B1] Olshanky S, Ault A (1986). The fourth stage of the epidemiologic transisiton: the age of delayed degenerative diseases. Milbank Quarterly.

[B2] Reddy K, Yusuf S (1998). Emerging epidemic of cardiovascular disease in developing countries. Ciruclation.

[B3] Omran A (2001). The epidemiologic transtion. A theory of the Epidemiology of population change. Bull World Health Organ.

[B4] Popkin B (2003). Dynamics of the nutrition transition and its implications for the developing world. Forum Nutr.

[B5] Yach D, Hawkes C, Gould C, Hofman K (2004). The global burden of chronic diseases: overcoming impediments to prevention and control. JAMA.

[B6] Fezeu L, Minkoulou E, Balkau B, Kengne AP, Awah P, Unwin N, Alberti GK, Mbanya JC (2006). Association between socioeconomic status and adiposity in urban Cameroon. Int J Epidemiol.

[B7] Kadiri S, Salako B (1997). Cardiovascular risk factors in middle aged Nigerians. East African Medical Journal.

[B8] Asfaw A (2005). The effects of obesity on doctor-diagnosed chronic diseases in Africa: empirical results from Senegal and South Africa. Journal of public health policy.

[B9] Bonita R, Beaglehole R (2007). Stroke prevention in poor countries: time for action. Stroke.

[B10] Kengne AP, Awah PK (2009). Classical Cardiovascular risk factors and all-cause mortality in rural Camroon. QJM.

[B11] Peer N, Steyn K, Bradshaw D, Norman R, Laubscher R, Dennison CR, Levitt NS, Nyo MT, Nel JH, Commerford P (2008). determinants of target organ damage in black hypertensive patients attending primary health care services in Cape Town: the Hi-Hi study. Am J Hypertension.

[B12] Unwin N, Setel P, Rashid S, Mugusi F, Mbanya J, Kitange H, Hayes L, Edwards R, Aspray T, Alberti K (2001). Noncommunicable diseases in sub-Saharan Africa: where do they feature in the health research agenda?. Bull World Health Organ.

[B13] Bonow R, Smaha L, Smith SCJr, Mensah GA, Lenfant C (2002). World Heart Day 2002: the international burden of cardiovascular disease: responding to the emerging blobal epidemic. Circulation.

[B14] Kengne AP, Amoah AGB, Mbanya JC (2005). Cardiovascular Complications of Diabetes Mellitus in Sub-Saharan Africa. Circulation.

[B15] Duda R, Kim M, Darko R, Adanu R, Seffah J, Anarfui J, Hill A (2007). Results of the Women's Health Study of Accra: assessment of blood pressure in urba women. Int J Cardiol.

[B16] Steyn K, Sliwa K, Hawken S, Commerford P, Onen C, Damasceno A, Ounpuu S, Yusuf S, INTERHEART Investigators in Afric (2005). Risk factors associated with myocardial infarction in Africa: the INTERHEART Africa study. Circulation.

[B17] Sande MA van der, Milligan PJ, Walraven GE, Dolmans WM, Newport M, Nyan OA, Banya WA, Thien T, Ward R, McAdam KP (2001). Geographical variation in prevalence of hypertension within The Gambia. Journal of human hypertension.

[B18] Sande MA van der (2003). Cardiovascular disease in sub-Saharan Africa: a disaster waiting to happen. Neth J Med.

[B19] Sande MA van der, Milligan PJ, Nyan OA, Rowley JT, Banya WA, Ceesay SM, Dolmans WM, Thien T, McAdam KP, Walraven GE (2000). Blood pressure patterns and cardiovascular risk factors in rural and urban Gambian communities. J Hum Hypertens.

[B20] Hoel D, Howard RB (1997). Hypertension: stalking the silent killer. Postgrad Med.

[B21] Addo J, Amoah AG, Koram KA (2006). The changing patterns of hypertension in Ghana: a study of four rural communities in the Ga District. Disease Ethnology.

[B22] Addo J, Smeeth L, Leon DA (2007). Hypertension In Sub-Saharan Africa: A Systematic Review. Hypertension.

[B23] Agyemang CO (2006). Rural and urban differences in blood pressure and hypertension in Ghana, West Africa. Public Health.

[B24] Agyemang C, Bruijnzeels MA, Owusu-Dabo E (2006). Factors associated with hypertension awareness, treatment, and control in Ghana, West Africa. J Hum Hypertens.

[B25] Agyemang C, Owusu-Dabo E (2008). Prehypertension in the Ashanti region of Ghana, West Africa: An opportunity for early prevention of clinical hypertension. Public Health.

[B26] Mufunda J, Mebrahtu G, Usman A, Nyarango P, Kosia A, Ghebbrat Y (2006). The prevalence of hypertension and its relationship with obesity: Results from a national blood pressure survey in Eritrea. J Hum Hypertens.

[B27] Adedoyin RA, Mbada CE, Balogun MO, Martins T, Adebayo RA, Akintomide A, Akinwusi PO (2008). Prevalence and pattern of hypertension in a semiurban community in Nigeria. Eur J Cardiovasc Prev Rehabil.

[B28] Ofuya Z (2007). The incidence of hypertension among a select population of adults in the Niger Delta region of Nigeria. Southeast Asian J Trop Med Public Health.

[B29] Lawoyin TO (2002). Prevalence of cardiovascular risk factors in an African, urban inner city community. West African journal of medicine.

[B30] Kaufman JS, Owoaje EE, Rotimi CN, Cooper RS (1999). Blood pressure change in Africa: case study from Nigeria. Hum Biol.

[B31] Poulter NR, Khaw K, Hopwood BEC, Mugambi M, Peart WS, Sever PS (1985). Determinants of blood pressure changes due to urbanisation: a longitudinal study. J Hypertens.

[B32] Steyn KBD, Norman R, Laubscher R (2008). Determinants and treatment of hypertension in South Africans: the first Demographic and Health Survey. S Afr Med J.

[B33] Mbanya JC, Ramiaya K, Jamison DT, Feachem RG, Makgoba MW, Bos ER, Baingana FK, Hofman KJ, Rogo KO (2006). Diabetes. Disease and Mortality in Sub-Saharan Africa.

[B34] International Diabetes Federation (2006). The Diabetes Declaration and Strategy for Africa. http://www.idf.org/webdata/docs/Diabetes%20Declaration%20&%20Strategy%20for%20Africa_full.pdf.

[B35] Beran D, Yudkin JS (2006). Diabetes care in sub-Saharan Africa. Lancet.

[B36] O WH, World Health Organization (1999). Diagnosis and Classification of Diabetes Mellitus. Definition, Diagnosis and Classification of Diabetes Mellitus and Its Complications.

[B37] Gill G, Mbanya JC, Ramaiya K, Tesfaye S (2009). A sub-Saharan African perspective of diabetes. Diabetologia.

[B38] Levitt NS, Katzenellenbogen JM, Bradshaw D, Hoffman MN, Bonnici F (1993). The prevalence and identification of risk factors for NIDDM in urban Africans in Cape Town, South Africa. Diabetes Care.

[B39] Mbanya JC, Ngogang J, Salah JN, Minkoulou E, Balkau B (1997). Prevalence of NIDDM and impaired glucose tolerance in a rural and an urban population in Cameroon. Diabetologia.

[B40] Elbagir M, Eltom MA, Elmahadi EMA, Kadam IMS, Berne C (1998). A high prevalence of diabetes mellitus and impaired glucose tolerance in the Danagla community in northern Sudan. Diab Med.

[B41] Motala AA, Omar MA, Pirie FJ (2003). Epidemiology of type 1 and type 2 diabetes in Africa. J Cardiovasc Risk.

[B42] Balde MN (2006). Associated tuberculosis and diabetes in Conkary, Guinea; prevalence and clinical chracateristics. The international journal of tuberculosis and lung disease.

[B43] Mollentze WF, Moore AJ, Steyn AF, Joubert G, Steyn K, Oosthuizen GM, Weich DJ (1995). Coronary heart disease risk factors in a rural and urban Orange Free State black population. S Afr Med J.

[B44] Baldé NMDI, Baldé MD, Barry IS, Kaba L, Diallo MM, Kaké A, Camara A, Bah D, Barry MM, Sangaré-Bah M, Maugendre D (2007). Diabetes and impaired fasting glucose in rural and urban populations in Futa Jallon (Guinea): prevalence and associated risk factors. Diabetes Metab.

[B45] Njelekela MA, Mpembeni R, Muhihi A, Mligiliche NL, Spiegelman D, Hertzmark E, Liu E, Finkelstein JL, Fawzi WW, Willett WC (2009). Gender-related differences in the prevalence of cardiovascular disease risk factors and their correlates in urban Tanzania. BMC Cardiovasc Disord.

[B46] Okafor CI, Fasanmade OA, Oke DA (2008). Pattern of dyslipidaemia among Nigerians with type 2 diabetes mellitus. Niger J Clin Pract.

[B47] Elnasri HA, Ahmed AM (2008). Patterns of lipid changes among type 2 diabetes patients in Sudan. East Mediterr Health J.

[B48] Otieno CF, Mwendwa FW, Vaghela V, Ogola EN, Amayo EO (2005). Lipid profile of ambulatory patients with type 2 diabetes mellitus at Kenyatta National Hospital, Nairobi. East Afr Med J.

[B49] Fezeu L, Balkau B, Kengne A, Sobngwi E, Mbanya J (2007). Metabolic syndrome in a sub-Saharan African setting: central obesity may be the key determinant. Atherosclerosis.

[B50] Eghan BA, Acheampong JW (2003). Dyslipidemia in outpatients at General Hospital in Kumasi, Ghana: cross-sectional study. Croat Med J.

[B51] Akpa MR, Agomouh DI, Alasia DD (2006). Lipid profile of healthy adult Nigerians in Port Harcourt, Nigeria. Niger J Med.

[B52] Chapman AR (2009). Globalization, human rights, and the social determinants of health. Bioethics.

[B53] BeLue R, Schreiner A, Taylor-Richardson K, Murray-Kolb L, Beard J (2008). What matters most: an investigation of predictors of perceived stress among young mothers in Khayelitsha. Health Care for Women International.

[B54] Olufemi O, Oluseyi M (2007). The urban poor and mobility stress in Nigerian cities. Environmental Research Journal.

[B55] Pike I, Williams S (2006). Incorporating psychosocial health into biocultural models: preliminary findings from Turkana women of Kenya. Am J Hum Biol.

[B56] Siervo M, Grey P, Nyan OA, Prentice AM (2006). Urbanization and obesity in the Gambia: a country in the early stages of the demographic transition. European journal of clinical nutrition.

[B57] Baum A, Garofalo JP, Yali AM (2006). Socioeconomic status and chronic stress: does stress account for SES effects on health?. Annals of the New York Academy of Sciences.

[B58] Levenstein S, Smith MW, Kaplan G (2001). Psychosocial predictors of hypertension in men and women. Arch Intern Med.

[B59] Yen I, Kaplan G (1996). Poverty area residence and changes in depression and perceived health status: evidence from the Alameda County Study. International Journal of Epidemiology.

[B60] Mfenyana K, Griffin M, Yogeswaran P, Modell B, Modell M, Chandia J (2006). Socio-economic inequalities as a predictor of health in South Africa - The Yenza cross-sectional. S Afr Med J.

[B61] Gulis G, Mulumba JA, Juma O, Kakosova BA (2003). Health status of people of slums in Nairobi, Kenya. Environmental Research.

[B62] Niakara A, Fournet F, Gary J, Harang M, Nebie L, Salem G (2007). Hypertension, urbanization, social and spatial disparities: a cross-sectional population-based survey in West African urban environment (Ouagadougou, Burkina Faso). Transactions of the Royal Society of Tropical Medicine and Hygiene.

[B63] Sobngwi E, Mbanya J, Unwin N, Porcher R, Kengne A, Fezeu L (2004). Exposure over the life course to an urban environment and its relation with obesity, diabetes, and hypertension in rural Cameroon. International Epidemiological Association.

[B64] Kaufman JS, Rotimi CN, Brieger WR, Oladokum MA, Kadiri S, Osotimehin BO, Cooper RS (1996). Determinants of Hypertension in West Africa: Contribution of anthropometric and dietary risk factors to urban-rural and socioeconomic gradients. American Journal of Epidemiology.

[B65] Malan LMN, Wissing MP, Seedat YK (2008). Coping with urbanization: a cardiometabolic risk? The THUSA study. Biol Psychol.

[B66] Agyemang C, Owusu-Dabo E, de Jonge A, Martins D, Ogedegbe G, Stronk K (2008). Overweight and obesity among Ghanaian residents in The Netherlands: how do they weigh against their urgan and rural counterparts in Ghana?. Public Health Nutr.

[B67] Assah F, Ekelund U, Brage S, Corder K, Wright A, Mbanya J, Wareham N (2009). Predicting physical activity energy expenditure using accelerometry in adults from sub-Sahara Africa. Obesity.

[B68] Kimokoti RW, Hamer DH (2008). Nutrition, health, and aging in sub-Saharan Africa. Nutr Rev.

[B69] Rotimi CN, Cooper RS, Ataman SL, Osotimehin B, Kadiri S, Muna W, Kingue S, Fraser H, McGee D (1995). Distribution of anthropometic variables and the prevalence of obesity in populations of West African origin: The international collaborative study on hypertension in blacks (ICSHIB). Obesity research.

[B70] Kamadjeu RM, Edwards R, Atanga JS, Kiawi EC, Unwin N, Mbanya JC (2006). Anthropometry measures and prevalence of obesity in the urgan adult population of Cameroon: an update from the Cameroon Burden of Diabetes Baseline Survey. BMC Public Health.

[B71] Abubakari AR, BHopal RS (2008). Systematic review on the prevalence of diabetes, overweight/obesity and physical inactivity in Ghanaians and Nigerians. Public Health.

[B72] Abubakari AR, Lauder W, Agyemang C, Jones M, Kirk A, Bhopal RS (2008). Prevalence and time trends in obesity among adult West African populations: a meta-analysis. Obes Rev.

[B73] Ntandou G, Delisle H, Agueh V, Fayomi B (2009). Abdominal obesity explains the positive rural-urban gradient in the prevalence of the metabolic syndrome in Benin, West Africa. Nutr Res.

[B74] Longo-Mbenza B, Mambune HF, Kasiamm JB, Vita EK, Fuele SM, Nsenga JN, Mabwa L, Nzuzi V (2007). Relationship between waist circumference and cholesterol in Central Africans with congestive heart failure. West Afr J Med.

[B75] Bourne LT, Lambert EV, Steyn K (2002). Where does the black population of South Africa stand on the nutrition transition?. Public Health Nutr.

[B76] Joubert J, Norman R, Lambert EV, Groenewald P, Schneider M, Bull F, Bradshaw D (2007). South African Comparative Risk Assessment Collaborating Group. Estimating the burden of disease attributable to physical inactivity in South Africa in 2000. S Afr Med J.

[B77] Fezeu L, Assah F, Balkau B, Mbanya D, Kengne A, Awah P, Mbanya J (2008). Ten-year changes in central obesity and BMI in rural and urban Cameroon. Obesity.

[B78] Christensen DL, Eis J, Hansen AW, Larsson MW, Mwaniki DL, Kilonzo B, Tetens I, Boit MK, Kaduka L, Borch-Johnsen K (2008). Obesity and regional fat distribution in Kenyan populations: impact of ethnicity and urbanization. Ann Hum Biol.

[B79] Siminialayi I, Emem-Chioma P, Dapper D (2008). The prevalence of obesity as indicated by BMI and waist circumference among Nigerian adults attending family medicine clinics as outpatients in Rivers State. Niger, J Med.

[B80] Dugas LR, Carstens MA, Ebersole K, Schoeller DA, Durazo-Arvizu RA, Lambert EV, Luke A (2009). Energy expenditure in young adult urban informal settlement dwellers in South Africa. Eur J Clin Nut.

[B81] Holdsworth M, Gartner A, Landais E, Maire B, Delpeuch F (2004). Perceptions of healthy and desirable body size in urban Senegalese women. International journal of obesity.

[B82] Holdsworth M, Delpeuch F, Landais E, Gartner A, Eymard-Duvernay S, Maire B (2006). Knowledge of dietary and behaviour-related determinants of non-communicable disease in urban Senegalese women. Public Health Nutr.

[B83] Duda R, Jumah N, Hill A, Steffah J, Birtwum R (2007). Assessment of the ideal body image of women in Accra, Ghana. Trop Doct.

[B84] Duda R, Jumah N, Hill A, Seffah J, Biritwum R (2006). Interest in healthy living outweighs presumed cultural norms for obesity for Ghanaian women. Health Qual Life Outcomes.

[B85] Townsend M, Peerson J, Love B, Achterberg C, Murphy S (2001). Food insecurity is positively related to overweight in women. J Nutr.

[B86] Chaput JP, Gilbert JA, Tremblay A (2007). Relationship between food insecurity and body composition in Ugandans living in urban Kampala. J Am Diet Assoc.

[B87] Ormel J, Von Korff M, Burger H, Scott K, Demyttenaere K, Huang Y, Posada-Villa J, Pierre Lepine J, Angermeyer MC, Levinson D (2007). Mental disorders among persons with heart disease-results from World Mental Health surveys. Gen Hosp Psychiatry.

[B88] Laabes EP, Thacher TD, Okeahialam BN (2008). Risk factors for heart failure in adult Nigerians. Acta Cardiol.

[B89] Puepet F, Ohwovoriole A (2008). Prevalence of risk factors for diabetes mellitus in a non-diabetic population in Jos, Nigeria. Niger J Med.

[B90] Christensen DL, Friss H, Mwaniki DL, Kilonzo B, Boit MK, Omondi B, Kaduka L, Borch-Johnsen K (2009). Prevalence of glucose intolerance and associated risk factors in rural and urban populations of different ethnic groups in Kenya. Diabetes Res Clin Pract.

[B91] Schneider M, Norman R, Parry C, Bradshaw D, Pluddemann A (2000). South African comparative risk assessment collaborating group. Estimating the burden of disease attributable to alcohol use in South Africa. S Afr Med J.

[B92] Schneider M, Bradshaw D, Steyn K, Norman R, Laubscher R (2009). Poverty and non-communicable diseases in South Africa. Scand J Public Health.

[B93] Verdier F, Fourcade L (2007). Changes in cardiovascular risk factors in developing countries. Med Trop.

[B94] Townsend L, Flisher AJ, Gilreath T, King G (2006). A systematic literature review of tobacco use among adults 15 years and older in sub-Saharan Africa. Drug and Alcohol Dependence.

[B95] Seck M, Diouf I, Acouetey L, Wade K, Thiam M, Diatta B (2007). Profile of patients admitted for myocardial infarction at the emergency reception facility of Principal Hospital in Dakar, Senegal. Med Trop.

[B96] Seyoum B, Abdulkadir J, Berhanu P, Feleke F, Worku Y, Ayana G (1999). Profile of coronary artery risk factors in Ethiopian diabetic patients. East Afr Med J.

[B97] Awah PK, Kengne AP, Fezeu LL, Mbanya JC (2008). Perceived risk factors of cardiovascular diseases and diabetes in Cameroon. Health Educ Res.

[B98] Kiawi E, Edwards R, Shu J, Unwin N, Kamadjeu R, Mbanya JC (2006). Knowledge, attitudes, and behavior relating to diabetes and its main risk factors among urban residents in Cameroon: a qualitative survey. Ethn Dis.

[B99] Pakenham-Walsh N, Bukachi F (2009). Information needs of health care workers in developing countries: a literature review with a focus on Africa. Hum Resour Health.

[B100] Motala AA (2002). Diabetes trends in Africa. Diabetes Metab Res Rev.

[B101] Whiting DR, Hayes L, Unwin NC (2003). Diabetes in Africa. Challenges to health care for diabetes in Africa. J Cardiovasc Risk.

[B102] Beran D, Yudkin JS, de Courten M (2005). Access to care for patients with insulin-requiring diabetes in developing countries. Case studies of Mozambique and Zambia. Diabetes Care.

[B103] Goudge J, Gilson L, Russel lS, Gumede T, Mills A (2009). Affordability, availability and acceptability barriers to health care for the chronically ill: longitudinal case studies from South Africa. BMC Health Serv Res.

[B104] Watkins P, Alemu S (2003). Delivery of diabetes care in rural Ethiopia: an experience from Gondar. Ethiop Med J.

[B105] Ohene-Buabeng K, Matowe L, Plange-Rhule J (2004). Unaffordable drug prices: the major cause of non-compliance with hypertension medication in Ghana. J Pharm Pharm Sci.

[B106] Thorogood M, Connor MD, Hundt GL, Tollman SM (2007). Understanding and managing hypertension in an African sub-district: a multidisciplinary approach. Scand J Public Health Suppl.

[B107] de-Graft Aikins A (2005). Healer shopping in Africa: new evidence from rural-urban qualitative study of Ghanaian diabetes experiences. BMJ.

[B108] Winston C, Patel V (1995). Use of traditional and orthodox health services in urban Zimbabwe. Int J Epidemiol.

[B109] Pinkoane MG, Greeff M, Koen MP (2008). Policy makers' perceptions and attitudes regarding incorporation of traditional healers into the national health care delivery system. Curationis.

[B110] Pinkoane MG, Greeff M, Williams MJ (2005). The patient relationship and therapeutic techniques of the South Sotho traditional healer. Curationis.

[B111] Abo KA, Fred-Jaiyesimi AA, Jaiyesimi AE (2008). Ethnobotanical studies of medicinal plants used in the management of diabetes mellitus in South Western Nigeria. J Ethnopharmacol.

[B112] Risenga P, Botha A, Tjallinks J (2007). Shangaan patients and traditional healers management strategies of hypertension in Limpopo Province. Curationis.

[B113] Hundt GL, Stuttaford M, Ngoma B, SASPI Team (2004). The social diagnostics of stroke-like symptoms: healers, doctors and prophets in Agincourt, Limpopo Province, South Africa. J Biosoc Sci.

[B114] Sengwana M, Puoane T (2004). Knowledge, beliefs and attitudes of community health workers about hypertension in the Cape Peninsula, South Africa. Curationis.

[B115] Peltzer K, Khoza LB, Lekhuleni ME, Madu SN, Cherian VI, Cherian L (2001). Concepts and treatment for diabetes among traditional and faith healers in the northern province, South Africa. Curationis.

[B116] Niang C (1994). The Dimba of Senegal: A support group for women. Reproductive Health Matters.

[B117] Dutta-Bergman M (2004). The unheard voices of Santalis: Communicating about health from the margins of India. Communication Theory.

[B118] Achebe C (1958). Things fall apart.

[B119] de-Graft Aikins A (2004). Strengthening quality and continuity of diabetes care in rural ghana: a critical social psychological approach. J Health Psychol.

[B120] Awah P, Unwin N, Phillimore P (2009). Diabetes Mellitus: Indigenous naming, indigenous diagnosis and self-management in an African setting: the example from Cameroon. BMC Endocr Disord.

[B121] Kagee A, Le Roux M, Dick J (2007). Treatment adherence among primary care patients in a historically disadvantaged community in South Africa: a qualitative study. J Health Psychol.

[B122] Airhihenbuwa C (2007). Healing our differences - the crisis of global health and the politics of identity. Lanham, Maryland.

[B123] Airhihenbuwa CO (1995). Health and Culture: Beyond the Western pardigm.

[B124] Airhihenbuwa C (1999). Of culture and multiverse: renouncing "the universal truth" in health. Journal of Health Education.

[B125] Ijarotimi O, Keshinro O (2008). Nutritional knowledge, nutrients intake and nutritional status of hypertensive patients in Ondo State, Nigeria. Tansania Journal of Health Research.

[B126] Sharma S, Mbanya J, Cruickshank K, Cade J, Tanya A, Cao X, Hurbos M, Wong M (2007). Nutritional composition of commonly consumed composite dishes from the Central Province of Cameroon. International Journal of Food Sciences and Nutrition.

[B127] Fajans J (1988). The transformative value of food: a review essay. Food and Foodways.

[B128] Boeck F (1994). 'When hunger goes around the land:' hunger and food among the Aluund of Zaire. Man.

[B129] Okoror TA, Airhihenbuwa CO, Shisana O, Zungu-Dirwayi N, Smith E, Brown D, Louw J (2008). "My mother told me I must not cook anymore" - Food, Culture, and the Contexts of HIV and AIDS related Stigma in Three Communities in South Africa. International Quarterly of Community Health Education.

[B130] Gwede C, McDermott R (1992). AIDS in Sub-Saharan Africa: Implications of health education. AIDS Education and Prevention.

